# Dimethyl fumarate dosing in humans increases frataxin expression: A potential therapy for Friedreich’s Ataxia

**DOI:** 10.1371/journal.pone.0217776

**Published:** 2019-06-03

**Authors:** Mittal Jasoliya, Francesco Sacca, Sunil Sahdeo, Frederic Chedin, Chiara Pane, Vincenzo Brescia Morra, Alessandro Filla, Mark Pook, Gino Cortopassi

**Affiliations:** 1 Department of Molecular Biosciences, School of Veterinary Medicine, University of California, Davis, California, United States of America; 2 Department of Neurosciences, Odontostomatological and Reproductive Sciences, University Federico II, Naples, Italy; 3 Department of Molecular and Cellular Biology, University of California, Davis, California, United States of America; 4 Department of Life Sciences, College of Health & Life Sciences, Brunel University London, Uxbridge, United Kingdom; Universidad Nacional de Rosario, ARGENTINA

## Abstract

Friedreich’s Ataxia (FA) is an inherited neurodegenerative disorder resulting from decreased expression of the mitochondrial protein frataxin, for which there is no approved therapy. High throughput screening of clinically used drugs identified Dimethyl fumarate (DMF) as protective in FA patient cells. Here we demonstrate that DMF significantly increases frataxin gene (*FXN*) expression in FA cell model, FA mouse model and in DMF treated humans. DMF also rescues mitochondrial biogenesis deficiency in FA-patient derived cell model. We further examined the mechanism of DMF's frataxin induction in FA patient cells. It has been shown that transcription-inhibitory R-loops form at GAA expansion mutations, thus decreasing *FXN* expression. In FA patient cells, we demonstrate that DMF significantly increases transcription initiation. As a potential consequence, we observe significant reduction in both R-loop formation and transcriptional pausing thereby significantly increasing *FXN* expression. Lastly, DMF dosed Multiple Sclerosis (MS) patients showed significant increase in *FXN* expression by ~85%. Since inherited deficiency in FXN is the primary cause of FA, and DMF is demonstrated to increase *FXN* expression in humans, DMF could be considered for Friedreich's therapy.

## Introduction

Friedreich’s ataxia (FA) is caused by inheritance of GAA tri-nucleotide expansions and reduced expression the mitochondrial protein frataxin. FA is an ultimately lethal neurodegenerative disease for which there is currently no approved therapy [1]. All pathophysiological consequences, severity and age of onset of FA are directly related to the extent of frataxin deficiency, greater the frataxin deficiency, worse the outcome [2–5]. Common symptoms associated with this disease include loss of muscle coordination, cardiomyopathy, hearing defect and diabetes [6,7].

How deficiency of the mitochondrial protein frataxin triggers the FA pathomechanism is still unclear, however the best evidence for frataxin's physiological role is that it supports mitochondrial iron-sulfur cluster synthesis [8,9]. Recently we demonstrated that frataxin deficiency in FA patient cells, FA mice, and FA human patients causes a mitochondrial biogenesis defect [10], so it is possible that a primary defect in iron-sulfur clusters (which are essential for several mitochondrial enzyme complexes) causes the mitochondrial biogenesis defect that ultimately triggers the disease.

To identify therapeutic strategies we screened 1,600 drugs of known safety profiles that are currently being used for other indications in humans [11]. Dimethyl fumarate (DMF) provided dose-dependent protection in cell-based screening. Chemically, DMF is methyl ester of fumaric acid and is currently used to treat Multiple Sclerosis (MS) and Psoriasis [12]. Here, we aim to analyze effect of DMF on Frataxin gene (*FXN*) expression in patient derived lymphoblast cell model, mice model YG8 and in blood lymphocytes from humans dosed with DMF.

We demonstrate that DMF dose-dependently increases *FXN* expression both in FA patient derived lymphoblast cells and in mouse *in vivo*. At the molecular level, inherited GAA repeats are thought to repress *FXN* expression through the formation of thermodynamically stable R-loop structure composed of an RNA-DNA hybrid and a displaced DNA single strand [13,14]. Presence of R-loop at expanded GAA site can result in stalling of RNA polymerase and premature transcription termination [15–17]. Mechanistically, we confirm that there are increased R-loops at the GAA repeat regions in the *FXN* gene of patient derived lymphoblastoid cell line. We further demonstrate that DMF significantly increases *FXN* expression in FA cells by increasing transcription initiation which potentially decreases R-loop enrichment and further reduces transcriptional stalling at GAA pause sites as demonstrated here. Because deficiency of mitochondrial frataxin is the primary cause of FA, and DMF significantly increases *FXN* expression in various models, we suggest that DMF could be considered as a potential therapy for FA.

## Results

### DMF increases FXN expression *in vitro* and *in vivo* in FA cells and mice models

**Fig 1:**
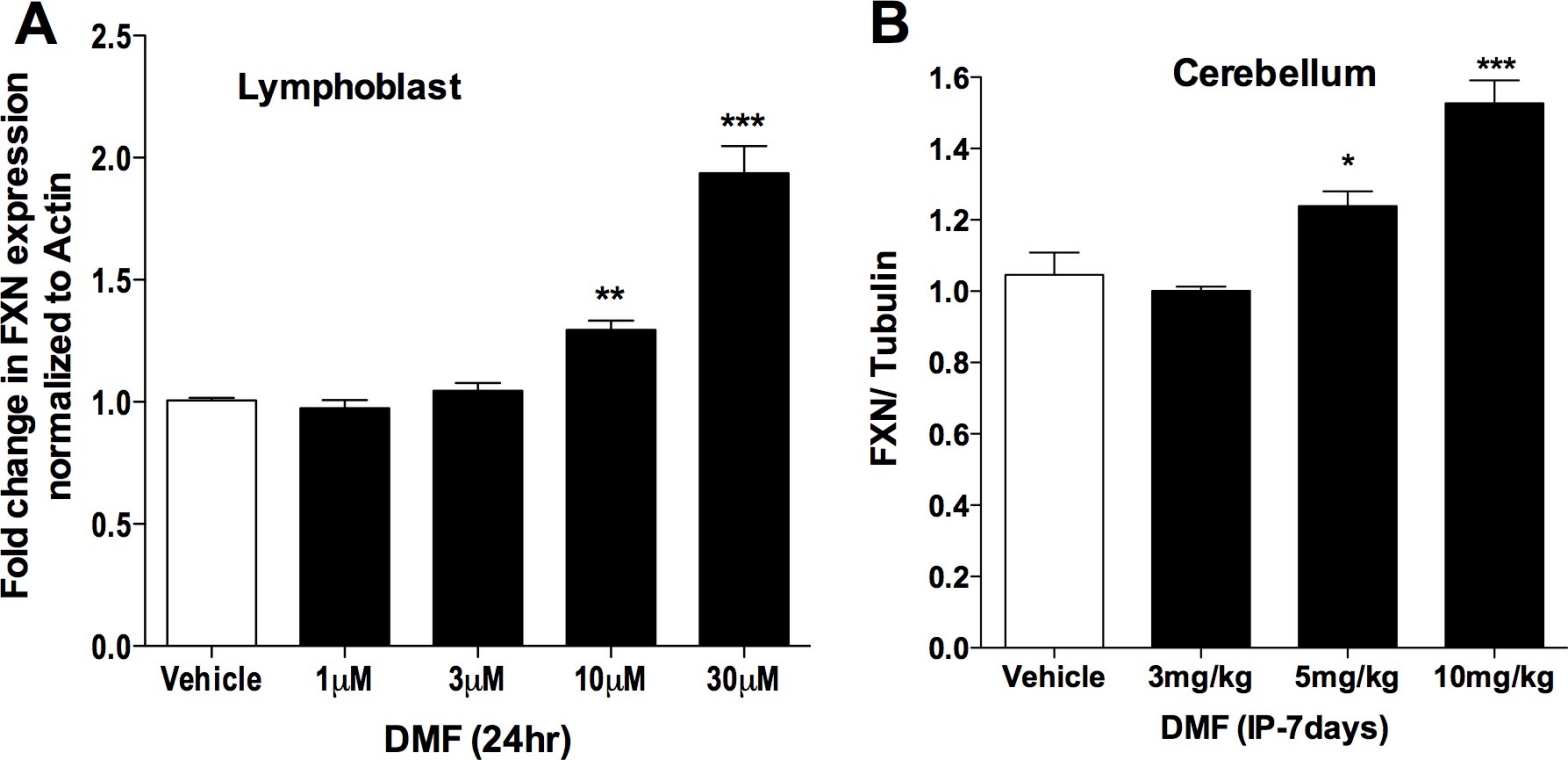
DMF dose dependently increases FXN expression *in vitro* in various patient derived lymphoblast cell models and *in vivo* in YG8 mice model of FA. (A) Lymphoblast cells (GM14518, GM15850, GM16214, GM16216 and GM16220) were treated with 0.01% DMSO vehicle, 1,3,10 or 30 μM DMF for 24hr. RNA was extracted and *FXN* expression was measured by qRT-PCR as FXN normalized to Actin. Vehicle, n = 50; 1μM, n = 3; 3μM, n = 20; 10μM, n = 24; 30μM, n = 42. (B) Mice were IP dosed with 3,5 and 10 mg/kg DMF for 7 day. Protein was extracted from Cerebellar tissue and measured using Western Blot analysis as Frataxin normalized to Tubulin or Actin. (n = 4; each group). Bars represent averages ± standard deviations (p<0.05*, p<0.01**, p<0.001***).

To study the effect of DMF on *FXN* expression we treated patient-derived FA lymphoblast cells GM14518, GM15850, GM16214, GM16216 and GM16220 that has different number of GAA repeats (S1 Table) with 1, 3, 10, 30 and 100 μM DMF for 24 hr. *FXN* mRNA expression was measured by qRT-PCR. We observed dose dependent increase in *FXN* mRNA expression with significant increase at 10 μM and 30 μM DMF concentration by 25% and 93% respectively, compared to their respective vehicle treated control (Fig 1A). The 100 μM dose was toxic and triggered significant cell death and very low RNA yields. We also confirmed increase in *FXN* expression at protein level with DMF treatment in lymphoblast cells (S1 Fig). Overall, DMF significantly increased *FXN* expression in various patient derived lymphoblast cell models.

To further determine if *in vitro* effects of DMF on FXN expression were translated *in vivo* in FA YG8 mice model, mice (n = 4) were intra-peritoneally (IP) dosed with DMF at 0, 3, 5 and 10mg/kg concentration for 7 days. Cerebellum, which is a primary tissue affected in FA was collected and frataxin protein level was measured by western blot. Consistently, we observed significant dose-dependent increase in frataxin level by 23% and 52% respectively in cerebellum of animal dosed with 5 and 10 mg/kg DMF compared to vehicle treated control animals (Fig 1B). Few representative images of frataxin western-blot from DMF treated YG8 mice model cerebellum is shown in (S2A & S2C Fig). Increase in frataxin was also observed in spleen of DMF treated YG8 mice model (S2B Fig) and cerebellum of DMF treated KIKO mice model (S2D Fig). Overall, DMF increases frataxin protein in the cerebellar tissue of various FA mice models, which is the target tissue affected in FA.

### DMF increases FXN expression *in vivo* in MS patients

12 controls and 27 MS patients were recruited from Multiple Sclerosis Center of the Federico II University of Naples. Of these 27 MS patients, 14 were treated with DMF and 13 with Fingolimod (FTY). Blood was collected before and after 3 months of treatment and PBMCs were isolated to determine *FXN* mRNA expression.

**Fig 2:**
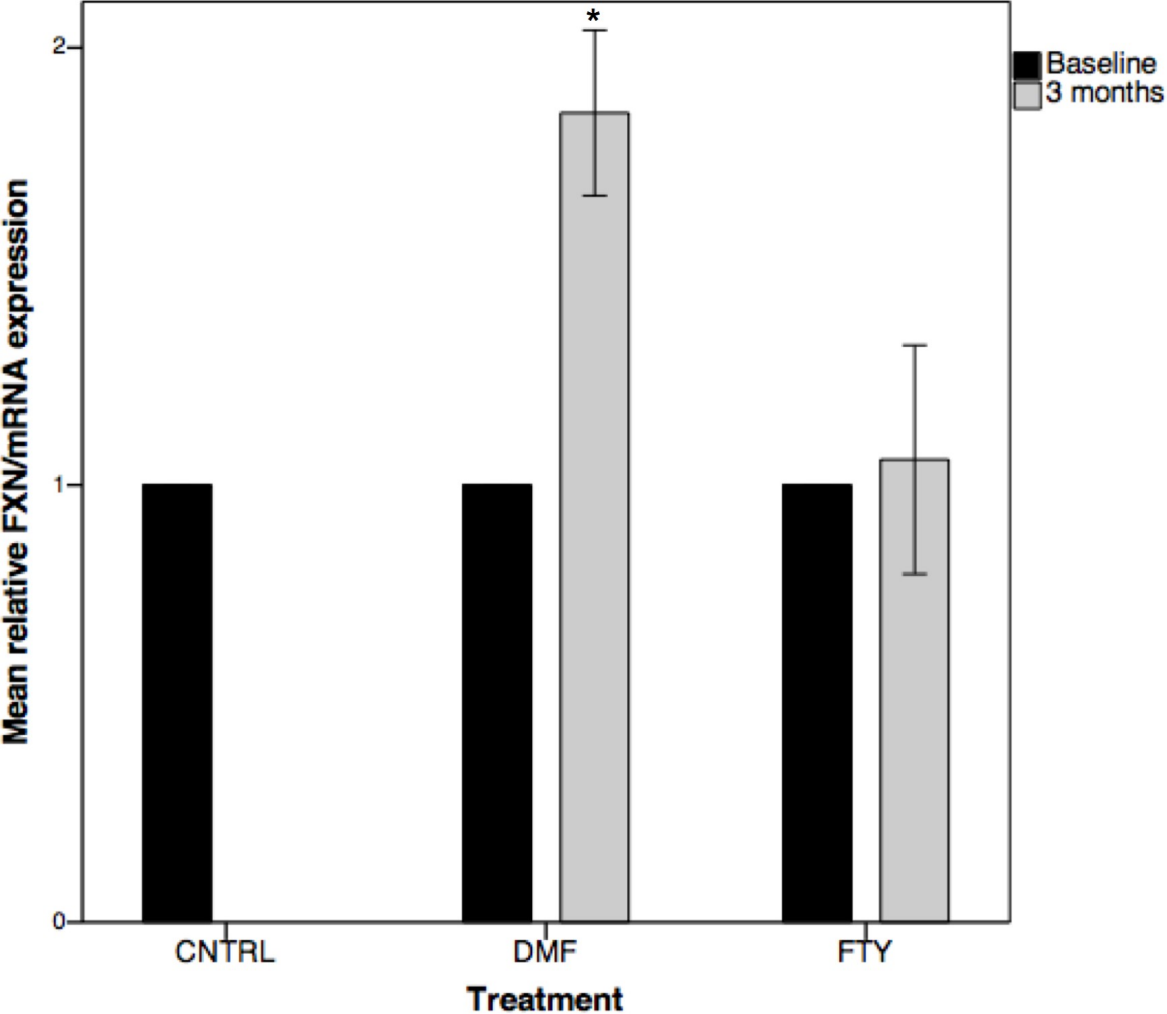
DMF increases FXN expression in peripheral blood mononuclear cells (PBMC’s) when dosed at 480mg/day *in vivo* in humans. 27 MS patients were recruited and 14 were treated with DMF and 13 with FTY for 3 months. Blood was drawn post treatment, PBMCs were isolated and FXN mRNA expression was measured pre and post treatment by qRT-PCR. Baseline values of FXN/mRNA expression were set at 1 and 3 months values are shown relative to baseline values. CNTRL = Controls (n = 12); DMF = Dimethylfumarate (n = 14); FTY = Fingolimod (n = 13);. Bars represent median ± error standard mean (p<0.05*).

Baseline levels of *FXN* mRNA were similar between controls and MS. We observed significant 85.1% increase in *FXN* expression in 3 months DMF treated MS patients. However, *FXN* expression was not much affected in FTY treated patient patients (Fig 2). Overall, consistent with cell and mice model, we observed a significant increase in FXN mRNA expression in peripheral blood lymphocytes of DMF treated MS patient.

### DMF induces transcription initiation and reduces transcriptional 'pausing' in mutant FXN gene

**Fig 3:**
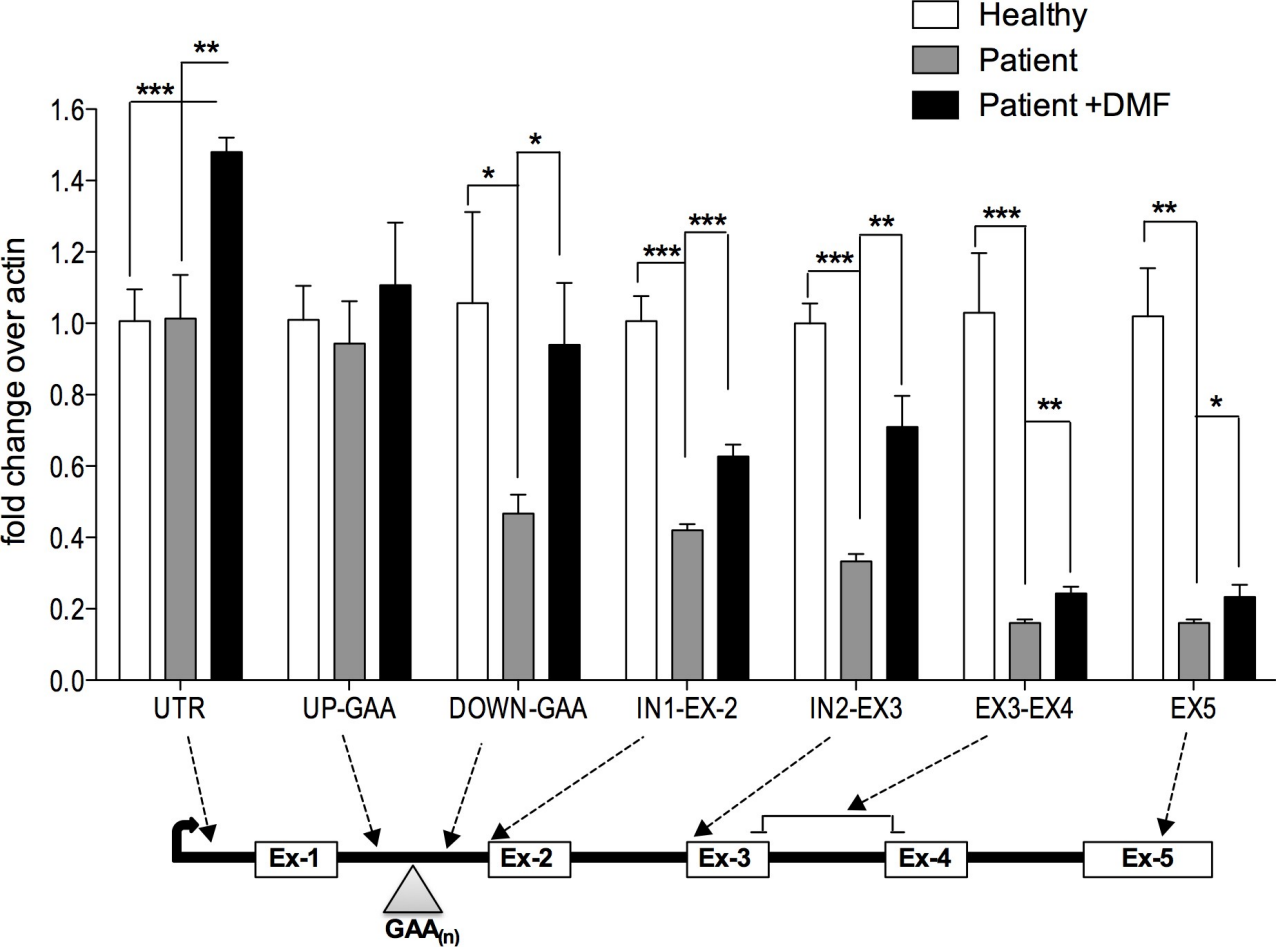
DMF induces both increased transcription initiation and procession at GAA repeat pause site of FXN. FXN pre-mRNA transcription was traced my qRT-PCR in healthy lymphoblast (GM-13068), patient derived lymphoblast (GM-14518) and in patient lymphoblast cells (GM14518) treated with 30uM DMF for 24 hr. Bars represent averages ± standard deviations (n = 3, each group). Bars represent averages ± standard deviations (p<0.05*, p<0.01**, p<0.001***).

The expanded GAA repeats that results in decreased FXN expression appear to do so by multiple mechanisms. In particular, GAA repeats can cause RNA polymerase 'pausing', which may be the result of co-transcriptional R-loop structures [18,19]. We studied the consequences of DMF dosing on FXN pre-mRNA transcription by qRT-PCR in patient derived lymphoblast cell line GM-14518 with ~900 GAA repeats by using primers (S2 Table) that are designed to follow RNA synthesis along the of *FXN* pre-mRNA (Fig 3). FXN pre-mRNA transcription profile of patient cell line at different loci was compared to healthy individual derived lymphoblast cell line GM-13068.

Consistent with a prior study [20], we observed 50% decrease in RNA transcript level in patient cells, just downstream of the GAA repeats, consistent with the idea that GAA repeats cause a 'stalling' of RNA polymerase in this region, resulting in decreased FXN expression. We observed additional transcriptional reduction along the transcript such that RNA levels were 60% lower than controls near Intron2-Exon3 and further reduced by 85% near exon 5, which is representative of mature FXN transcript (Fig 3).

Interestingly, when we treated the same GM14518 patient cells with 30 μM DMF treatment for 24 hour, we measured a 48% increase in transcript levels at the 5’UTR (Fig 3). In addition, transcript level was significantly higher at all the loci downstream of GAA pause site, increasing the level of mature transcript by ~146% compared to untreated patient cells, as measured by Ex3-Ex4 primers and Ex5 primers. Thus, DMF appears to increases FXN expression by two mechanisms: increased transcriptional initiation (Fig 3, UTR) and reversal of pausing (Fig 3, Down-GAA to Ex5).

### DMF reduces R-loop concentrations at mutant FXN pause sites

**Fig 4:**
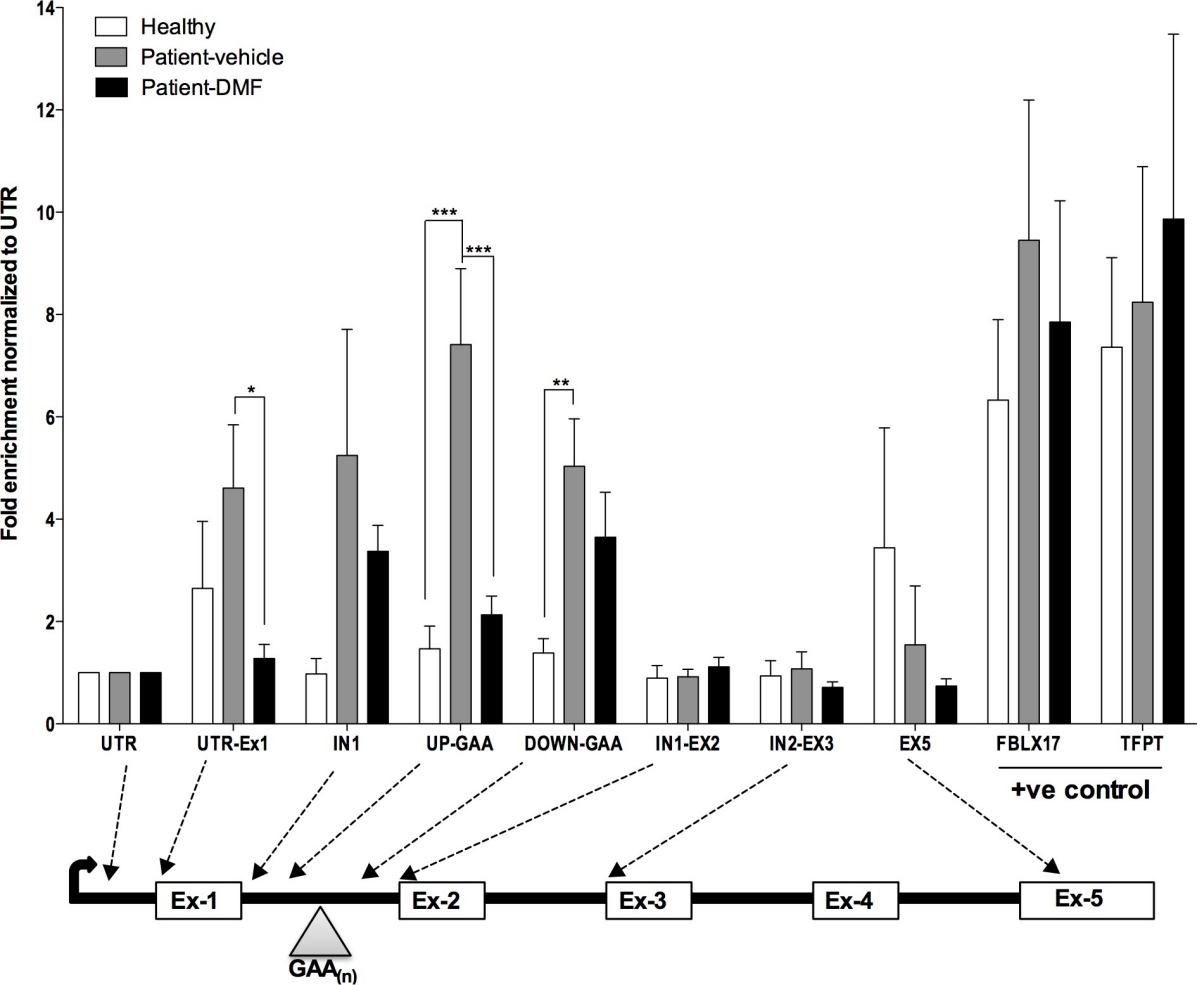
DMF reduces R-loop enrichment at GAA repeat pause site of FXN gene. R-loops were enriched using antibody specific for RNA-DNA hybrid and fold enrichment measured relative to input DNA by qPCR in healthy lymphoblast (GM-13068), patient derived lymphoblast (GM14518) and in patient lymphoblast cells (GM-14518) treated with 30uM DMF for 24 hr. Bars represent averages ± standard deviations (n = 6, each group, p<0.05*, p<0.01**, p<0.001***).

One of the mechanism proposed for RNA polymerase transcriptional pausing in mutant FXN is through the formation of R-loops structure [20]. R-loops formed over GAA repeats are thought to silence FXN expression through the recruitment of repressive histone marks and impairment of transcription caused by chromatin condensation. We tested whether DMF has any effect on R-loop formation using DNA-RNA hybrid immuno-precipitation (DRIP) to selectively enrich DNA-RNA hybrid containing R-loops in healthy lymphoblast cells GM-13068, patient derived lymphoblast cells GM-14518, and in patient cells treated with 30 μM DMF for 24 hr. DRIP was followed by qRT-PCR to detect R-loop enrichment at desired locations along the FXN gene. We observed a trend of increased R-loop enrichment at all loci near GAA repeats in FA patient cells, with significant enrichment at Up GAA and down GAA loci of FXN gene by 5 fold and 3.6 fold respectively (Fig 4) compared to healthy cells. Positive control gene loci, *FBLX17* and *TFPT* showed higher R-loop enrichments both in healthy cells and patient cells as expected. DMF treated patient cells showed an overall trend of decrease in R-loop enrichment compared to untreated patient cells with significant decrease at UTR-Ex1 and UP GAA loci by 3.6 and 3.4 fold respectively. Interestingly, R-loop enrichment at *TFPT* and *FBLX17* were not affected by DMF treatment. As a negative control for this experiment, DNA from patient derived lymphoblast cells was treated with RNase-H before DRIP. RNase-H treatment decreased R-loop enrichment in patient cells at loci near GAA repeats in *FXN* gene as well as decreased R-loop enrichment at positive control genes (S3 Fig).

Overall, DMF treatment reduced R-loop loads specifically around the GAA repeat region of *FXN*, suggesting that the increased transcription observed in DMF treated patient cells might be linked to the reduction of R-loop induced pausing.

### DMF increases mitochondrial biogenesis in patient derived fibroblast cells

**Fig 5:**
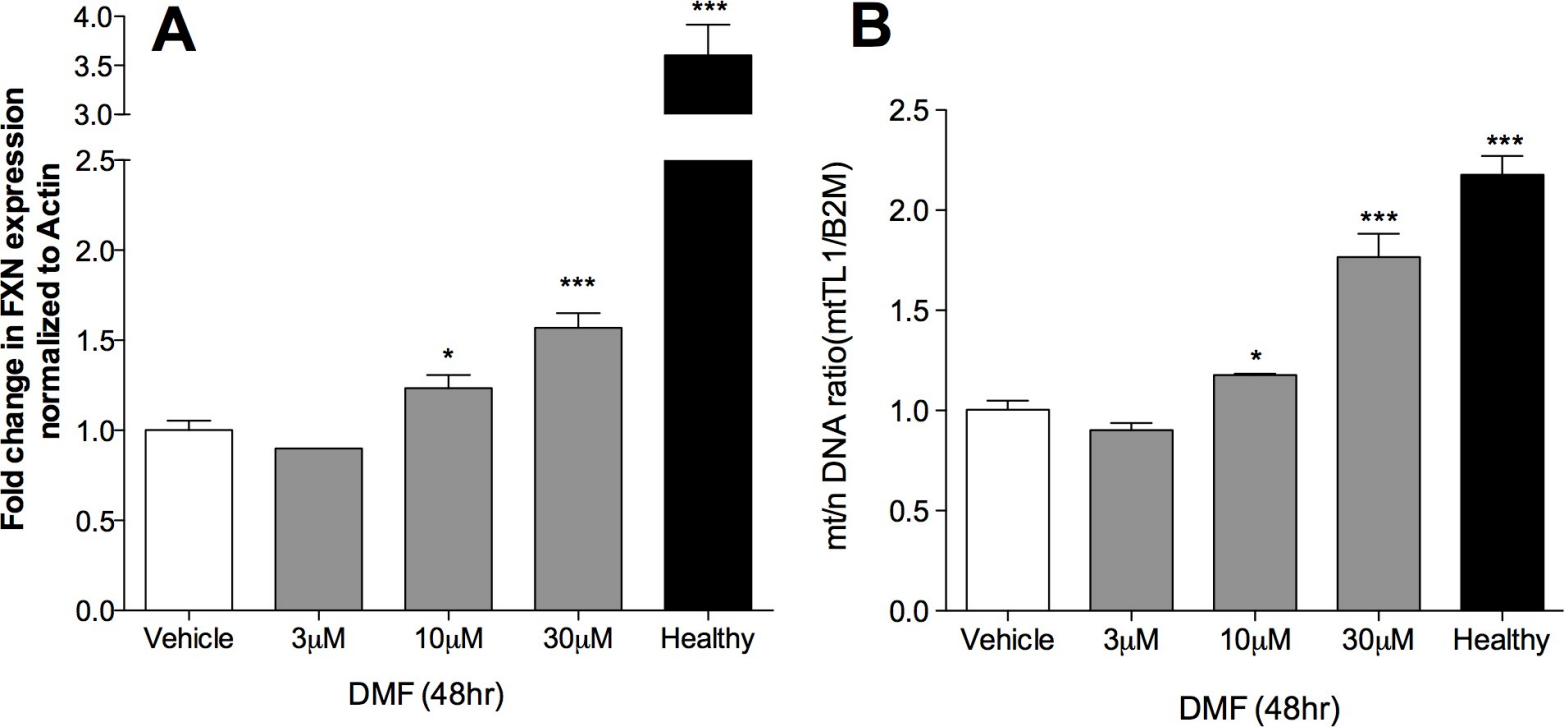
DMF increases *FXN* expression and mitochondrial biogenesis in 48 hr treated patient derived fibroblast cells. Cells were treated with 0.01% DMSO vehicle, 1,3,10 or 30 μM DMF for 48 hr (A) Frataxin mRNA expression measured by qPCR as FXN normalized to Actin. Vehicle, n = 8; 3μM, n = 1; 10μM, n = 4; 30μM, n = 8; Healthy, n = 3. (B) Mitochondrial DNA copy number was measured by q-PCR analysis as ratio of mitochondrial DNA over nuclear DNA; mt-TL1/B2M. Vehicle, n = 9; 3μM, n = 3; 10μM, n = 5; 30μM, n = 9; Healthy, n = 3. Bars represent averages ± standard deviations (p<0.05*, p<0.01**, p<0.001***).

We recently demonstrated a mitochondrial biogenesis defect in FA patient cells, FA mice, and FA patient blood lymphocyte tissue [10]. We also showed that the mitochondrial biogenesis defect was dependent on FXN concentration through siRNA knockdown and over-expression experiments. Furthermore, we showed that the mitochondrial biogenesis defect in FA patient blood was directly and significantly correlated with their blood FXN expression (Jasoliya et al., 2017). Given the highly energetic nature of neurons, this FXN-dependent mitochondrial biogenesis defect could be a significant contributor to the neurological patho-mechanism of Friedreich's ataxia. We treated FA fibroblasts GM-4078 with DMF for 48 hr. Total DNA was extracted and FXN expression, and mitochondrial copy number per cell was measured as mtDNA/nDNA ratio. Dose-dependent significant increases in mitochondrial copy number and FXN expression were observed. Mitochondrial copy number was significantly increased at 10 μM and 30 μM concentration by 20% and 76% respectively compared to vehicle treated control in 48hr DMF treated fibroblast cells (Fig 5B). Overall, DMF significantly increased mitochondrial copy number in FA patient derived primary cells. We recently demonstrated that DMF also increases mitochondrial biogenesis and gene expression in normal human fibroblasts, control mice, and in humans with Multiple Sclerosis (Hayashi Jasoliya). Since FXN deficiency triggers the mitochondrial biogenesis defect in cells, and DMF dose dependently increases FXN expression (Figs 1 & 5A), the increase in mitochondrial biogenesis we observed could potentially be the direct consequence of FXN induction.

## Discussion

### Effect of R-loops on FXN gene expression

GAA repeat expansions in the first intron of the *FXN* gene result in decreased FXN protein expression. Understanding the mechanistic relationship between increased GAA repeats and reduced *FXN* expression is important to therapeutic approaches for FA. Expanded GAA repeat generates a region with high G versus C strand asymmetry or GC skew, which thermodynamically favors re-invasion of the GA-rich transcript on the template DNA strand behind the advancing RNA polymerase, thus forming R-loops. R-loop formation has been shown to play a vital role in transcription termination in humans and could thus lead to pausing and/ or termination in the FXN intron1 as observed here (Fig 3).

An alternative, model for the effect of R-loop is the recruitment of repressive histone marks, leading to the compaction of chromatin [21–23]. Spreading of such repressive modifications to the promoter region could lead to the epigenetic silencing. Alternatively, chromatin compaction could lead to reduced elongation through the GAA repeats, as has been observed [24]. Thus R-loops could decrease FXN expression by causing premature transcription termination and/or decreasing transcription initiation or elongation because of chromatin compaction.

Recent evidence also hints at a possible mechanism by which repressive histone marks can be recruited by R-loops. Formation of R-loops at expanded GAA repeats leaves the GA-rich non-template DNA strand in a single stranded state that is accessible to the transcription machinery. This has been shown to trigger antisense RNA transcription [25]. The sense and antisense FXN transcripts may lead to formation of dsRNA thereby recruiting the RNA interference machinery and further decreasing the formation of mature FXN transcript by chopping of the existing mRNA using RISC complex and/or formation of repressive histone marks [23].

### DMF induces FXN expression by increased transcriptional initiation

We show that DMF induces FXN expression through increased transcriptional initiation. This potentially decreases transcriptional pausing (Fig 3) and reduces frequency of R-loop formation in mutant FXN genes. One possible cause of increased FXN initiation events is increased activation through the Nrf2 mediated mechanism, as described previously for Dyclonine. In this view, DMF which is a known Keap1/Nrf2 inducer, triggers Nrf2 binding to 3 Antioxidant Response Elements (AREs) upstream of the FXN gene, triggering increased transcriptional initiation [11]. Another possibility is that DMF, that drives the expression of mitochondrial biogenesis factors NRF1 and TFAM [26] drives the expression of all mitochondrial proteins including FXN as a consequence of increased mitochondrial biogenesis overall (Fig 5).

### DMF induces FXN and mitochondrial biogenesis at the concentrations it reaches in human dosing

Friedreich’s ataxia is a rare, autosomal recessive disease caused by depletion of mitochondrial protein FXN whose likely role is in Fe-S cluster biogenesis. Loss of FXN results in decrease in mitochondrial copy number [10], which primarily effects high energy demanding tissues, including dorsal root ganglia, dentate nuclei cerebellum, skeletal muscle and heart. Primary clinical features involve progressive loss of muscle coordination and balance, leading to gait and limb ataxia resulting in being wheel chair bound as the disease progresses. Cardiomyopathy is the leading cause of death in FA [27,28]. Currently there is no approved therapy for FA [29]. Here we show that DMF, approved for use in humans for multiple sclerosis and psoriasis, dose-dependently induces both FXN expression whose deficiency is the only cause of FA, and also induces mitochondrial biogenesis. Both the FXN induction and the mitochondrial biogenesis induction are happening at ~10 μM, which is close to the ~7 μM concentration reached by DMF's major bioactive metabolite MMF in 480mg dosing in humans.

### DMF as a potential therapy for FA

We show that DMF increases *FXN* expression by 93% in FA derived lymphoblast cell model at 30μM dose, by 52% in FA mice Yg8 at 10mg/kg dose and by 85% in MS patients treated for 3 months. There is a positive co-relation between FA patient FXN level in the blood to age of disease onset [30]. This modest increase in FXN is highly clinically relevant as increase in FXN by ~80% (Fig 2) has potential to delay the age of FA onset by many years.

It is also important to stress that *FXN* mRNA expression is reduced to 19.4% in FA patients, and to 53% in carriers, as compared to healthy controls [31]. Carriers are healthy individuals that discover their status only during genetic counseling, and their condition has never been linked to any neurological or cardiological abnormality. Therefore, the increase in FXN mRNA after DMF treatment may be effective in counteracting the pathological process of reduced FXN at the increase found after in vivo administration. Thus, DMF is unique, it is safe and well tolerated, it has more than 200,000 patients treated worldwide for Multiple Sclerosis and Psoriasis. This suggests that it should be considered as a therapy for Friedreich's ataxia.

## Materials and method

### Cell culture & drug treatment

Human lymphoblast cells: Healthy- GM13068; FA-patient derived- GM14518, GM15850, GM16216, GM16214 and GM16220 were grown in RPMI-1640 supplemented with 15% Fetal Bovine Serum. Human Fibroblast cells: Healthy- GM3440; FA patient derived- GM04078 were grown in DMEM supplemented with 12% Fetal Bovine Serum. For both cell types, Media was changed every two days. These cells were obtained from Coriell Institute for Medical Research Repository.

For *in vitro* drug treatment, 2x10^6^ cells were treated with 0.01% DMSO as vehicle control or 1, 3, 10, 30 and 100μM of dimethyl fumarate for 24 or 48 hrs. Post treatment cells are harvested, washed with PBS and processed for downstream analysis.

### Animal procedure

All animal procedures were approved by the ‘Institutional Animal Care and Use Committee’ at University of California, Davis. 50 mg/ml of DMF was dissolved in DMSO to make stock solution of DMF. Prior to injection, 0.5 mg/ml of working DMF solution (1:100 dilution) was made by diluting the stock solution into phosphate-buffered saline with 5% Tween-20 and 5% polyethylene glycol (Sigma-Aldrich, St. Louis, MO, USA). The mice were injected intraperitoneally every day for 7 days with 0,3,5and10 mg/kg of DMF. The mice were euthanized with CO_2_ followed by cervical dislocation and tissues were immediately removed then flash frozen with liquid nitrogen. Samples were stored in −80 °C until utilized for experiments.

Mice were randomly allocated to 4 dose groups- 0 (vehicle), 3, 5 and 10 mg/kg DMF dose. Sample size of 4 animals per dose group was determined by power calculation (Mean 1 = vehicle = 1, Mean 2 = 1.2 (20% Frataxin increase), Standard deviation (SD) = 0.1 = 10%, from calculated SDs of many technical replicates of FXN measurements of mouse cerebellum, 2 sided test, alpha = 0.05 and power of 0.80. Experimenter was no blinded to mice numbers during intra peritoneal injection, however after the westernblot was performed, images were quantified objectively by Li-Cor Odyssey software.

### Ethics and patients

The local Ethics Committee of the University Federico II of Naples, approved the study. The study was performed in accordance to the Declaration of Helsinki, European guidelines CPMP/ICH/135/95, and Italian law D.M.15/07/1997. All patients gave written informed consent before any activity linked to the study was started.

Patients were recruited from the Multiple Sclerosis Center of the Federico II University of Naples. There were 12 controls, and 27 MS patients. Of these 27 MS patients, 14 were treated with DMF and 13 with Fingolimod (FTY). Controls demographics were: age, gender. For DMF treated MS patients: age 42.84±11.9, gender M/F = 8/6, age at onset 29.2±9.8, disease duration 13.6±7.1, relapses in the previous year 0.6±0.7, EDSS 4.6±1.9. For FTY treated MS patients: age 45.9±23.3, gender M/F = 3/10, age at onset 34.2±20.9, disease duration 11.8±6.2, relapses in the previous year 0.5±0.7, EDSS 3.1±1.2.

Past medical history varied among MS patients. In the DMF cohort, three patients were treatment naïve for MS specific drugs, five had been previously treated with interferon beta-1a s.c., two with natalizumab, teo with interferon beta-1b s.c., one with interferon beta-1a i.m., one with FTY. In the FTY cohort, three were treatment naïve, three had been previously treated with interferon beta-1a s.c., two with natalizumab, two with interferon beta-1b s.c., three with interferon beta-1a i.m., one with FTY.

Patients were included if a decision to start a therapy with Dimethylfumarate (DMF) or fingolimod (FTY) had already been taken as part of clinical practice. We obtained samples on the day before treatment was started, and after 3 months of continuous DMF or FTY therapy. Patients treated with DMF received 120 mg, twice a day, for one week and 240 mg twice a day, for the remaining days until month 3. FTY patients were treated with 0.5 mg FTY for all 3 months. Healthy controls were recruited at our clinic through students and site personnel. Patients and controls were genotyped for the FXN gene expansions in order to exclude the presence of expanded alleles.

### RNA extraction and qRT-PCR

In *in vitro* cultured cells, RNA was exrtacted using RNAesay kit (QIAGEN). cDNA was then synthesized from mRNA with iScript cDNA Synthesis Kit (Bio-Rad Laboratories, Hercules, CA, USA) per manufacturer’s instruction in a C1000 Touch Thermal Cycler (Bio-Rad Laboratories, Hercules, CA, USA). A SensiFAST SYBR No-ROX Kit (Bioline, Taunton, MA, USA) was used to perform qPCR on the synthesized cDNA in a Roche Lightcycler 480 (Roche Diagnostics, Indianapolis, IN, USA). The second derivative of the amplification curve was used to determine the cycle threshold, and the data were analyzed by a delta delta CT calculation. Primer sets used in qPCR are listed in S2 Table.

For FXN gene expression in DMF treated patients, PBMCs were extracted from 30 mL of EDTA anticoagulated whole blood using Leucosep tubes (Greiner bio-one, Frickenhausen, Germany) and frozen at -80°C until analysis. Total mRNA was extracted from PBMCs using RIboPure RNA Purification Kit (ThermoFisher Scientific, Waltham, MA, USA) following manufacturer’s instructions. 500 ng mRNA was reversely transcribed using the one-step High Capacity RNA-to-cDNA Master Mix (ThermoFisher Scientific, Waltham, MA, USA) following manufacturer’s instructions in a total volume of 20μL. mRNA was quantified using a Gene Expression Assay for frataxin (Life Sciences, catalog n. Hs00175940_m1) and standardized by quantification of hypoxanthine phosphoribosyl-transferase 1 as a reference gene. Relative expression was calculated with the efficiency-calibrated model as previously described [32]

### Western blot

Cells/ mice tissues were lysed using lysis buffer (Cell Signalling) supplemented with a complete protease inhibitor cocktail (Roche Applied Science) and phenylmethylsulfonyl fluoride (Sigma-Aldrich Corp.). Tissues were further homogenized using 0.5 mm glass beads in a Bullet Blender high-throughput homogenizer (Next Advance, Inc.). After pelleting cellular debris by spinning at 16000 rpm at 4°C for 15 min, protein was quantified by Bradford assay. Detailed procedure for western blotting has been described previously [26]. In brief, 40–50 μg protein was added per lane of 4–12% Bis-Tris gels (Invitrogen Corp.). Primary antibodies were diluted in Odyssey blocking buffer (LI-COR Biosciences). Antibodies used included: anti-FXN (provided by Franco Taroni M.D., Istituto Besta) and anti-actin (#A2668, Sigma). Direct conjugated secondary antibodies (anti-rabbit IRdye800Cw and anti-mouse IRdye680 from LI-COR) were used to detect and quantify the signal of primary antibodies and imaged using a LI-COR Odyssey.

### Harvesting nucleic acids for DRIP

Cell pellets were washed with DPBS (Life Technologies) and resuspended in 4 mL of 10 mM Tris-HCl, 10 mM EDTA, 100 mM NaCl pH 8, lysed with 0.5% SDS, and digested with 400 units of Proteinase K (Thermo Fisher Scientific, Waltham, MA) at 37°C overnight. Cell lysates were then extracted once with 1 volume of equilibrated phenol pH 8 (USB, Cleveland, OH) and twice with 1 volume of chloroform (Sigma-Aldrich). DNA was precipitated with 1 volume of isopropanol and 300 mM sodium acetate, and was swirled out of solution with a glass shepherd's hook. The DNA pellet was washed twice by rinsing the hook with 400 μL of 70% ethanol, and was rehydrated in 10 mM Tris-HCl pH 8.

### DRIP-PCR

Harvested nucleic acids (∼50 μg) were digested using a restriction enzyme cocktail (20 units each of EcoRI, HindIII, BsrGI, PuvI, Ssp1) (New England Biolabs, Ipswich, MA; NEB) overnight at 37°C in 1× NEBuffer 2. Digests were cleaned by phenol and chloroform extraction followed by precipitation in isopropanol. The resulting fragmented DNA was pelleted at full speed (16,100× g) at 4°C and washed twice with 70% ethanol. Air-dried pellets were rehydrated in 10 mM Tris-HCl pH 7.5, 1 mM EDTA (TE).

We adapted the previously described DRIP protocol [13]. Six to eight μg of digested nucleic acids were diluted in 450 μL of TE, and 10 μL was reserved as input for qPCR. Fifty-two μL of 10× IP buffer was added for a final buffer concentration of 10 mM sodium phosphate, 140 mM sodium chloride, 0.05% Triton X-100, and 20 μL of S9.6 antibody (1 mg/ml; KeraFAST) The samples were incubated with the antibody at 4°C for 2 hours. This incubation and all wash steps were performed on a rotisserie mixer. 40 μL of Protein A/G Agarose beads (Pierce, Rockford, IL) was washed twice with 800 μL of 1× IP buffer for 5 minutes at room temperature. After adding agarose beads to each sample, they were incubated for 2 hours at 4°C. Each DRIP was then washed three times with 700 μL 1× IP buffer for 10 minutes per wash at room temperature. After the final wash, the agarose beads were resuspended in 250 μL of 1× IP buffer and incubated with 60 units of Proteinase K for 30 minutes at 50°C. Digested DRIP samples were then cleaned with phenol/chloroform extraction and isopropanol precipitation. Air-dried DRIP pellets were resuspended in 80 μL of 10 mM Tris-HCl pH 8.

We used 10 μL reactions with Sensi-FAST Lo-Rox 2× qPCR mix (Bioline, London, UK) to assay for genomic loci on FXN gene using Primer listed in the S2 Table. For each DRIP sample, 5 μL of the output and 5 μL of diluted input (1∶100) were assayed in triplicate. Fold enrichment for a given locus was calculated using the comparative Ct method.

### Materials

Dimethyl Fumarate was purchased from (Sigma-Aldrich, St. Louis, MO, USA) and stock solution was prepared in DMSO. Media for cell culture was obtained from (Corning, Inc., NY, USA) and Fetal Bovine Serum from Fetal Bovine Serum (JR Scientific, Woodland, CA).

### Statistics

Cell and Mice data are expressed as mean ± standard deviation. Relative comparison of data was performed with a t-test or one-way repeated measures analysis of Variance (ANOVA) using a host hoc Bonferroni correction. All statistical analysis was performed with Prism (GraphPad Software, La Jolla, USA) Differences were considered significant when p<0.05.

For Human dosing data, normality was tested with the Kolmogorov-Smirnov test. P values less than 0.05 were considered significant. The effect of DMF and FTY on frataxin expression was analyzed with a General Linear Model for repeated measures with *FXN* expression as the dependent variable at baseline and 3 months after treatment, and drug as a factor. Statistical analysis was performed with SPSS 23.0.0.2 running on MacOS 10.11.6.

## Supporting information

S1 Table Cell line and GAA repeat. (PDF)

S2 Table qPCR Primer list. (PDF)

S1 Fig Western Blot images to demonstrate increase in frataxin expression in DMF treated FA patient derived lymphoblast cell line. (PDF)

S2 Fig Western Blot images to demonstrate increase in frataxin expression in DMF treated FA mice models (YG8 and KIKO). (PDF)

S3 Fig RNAse-H treatment of patient cells eliminated R-loop enrichment. (PDF)
