# Supplementary material for: Dimethyl fumarate dosing in humans increases frataxin expression: A potential therapy for Friedreich’s Ataxia
Source: PLoS One. 2019 Jun 3;14(6):e0217776. doi: 10.1371/journal.pone.0217776 (PMC6546270; doi:10.1371/journal.pone.0217776)
Supplement: S1 Table — (PDF) [file pone.0217776.s001.pdf]

Cell line	GAA repeat	
	allele 1	allele 2
GM 14518	1122	925
GM15850	1030	650
GM16214	700	600
GM16216	500	200
GM16220	460	460
GM04078	541	420
GM16197	830	670
