# Supplementary material for: Dimethyl fumarate dosing in humans increases frataxin expression: A potential therapy for Friedreich’s Ataxia
Source: PLoS One. 2019 Jun 3;14(6):e0217776. doi: 10.1371/journal.pone.0217776 (PMC6546270; doi:10.1371/journal.pone.0217776)
Supplement: S2 Table — (PDF) [file pone.0217776.s002.pdf]

**Primers**	**Forward (5' --> 3')**	**Reverse (5'-->3')**
UTR	CAGTAAGCCAGGACCACACC	AGACGGGTTTCACCGTGTTA
UTR-Ex1	AGGAACATGCCCTGTCCA	TGTGTGACCAGAGGGCAAG
In-1	AAACTGACCCGACCTTTATTCCA	GGAATCCCCCAAGGTCACA
UP GAA	GAAACCCAAAGAATGGCTGTG	TTCCCTCCTCGTGAAACACC
DOWN-GAA	CTGGAAAAATAGGCAAGTGTGG	CAGGGGTGGAAGCCCAATAC
In1-Ex2	AGCACTCGGTTACAGGCACT	GCCCAAAGTTCCAGATTTCC
In2-Ex3	GGTAATCATGTTTTGGGTTTTGTGC	AGTCCTCAAACGTGTATGGCTTGTC
Ex3-Ex4 (mature FXN transcript)	CCTTGCAGACAAGCCATACA	GGTCCACTGGATGGAGAAGA
Ex5 (mature FXN transcript)	ATCTTCTCCATCCAGTGGACCT	GCTGGGCATCAAGCATCTTTT
mt-TL1	CACCCAAGAACAGGGTTTGT	TGGCCATGGGTATGTTGTTA
B2M	TGCTGTCTCCATGTTTGATGTATCT	TCTCTGCTCCCCACCTCTAAGT
