# Supplementary figures and images for: Dimethyl fumarate dosing in humans increases frataxin expression: A potential therapy for Friedreich’s Ataxia
Source: PLoS One. 2019 Jun 3;14(6):e0217776. doi: 10.1371/journal.pone.0217776 (PMC6546270; doi:10.1371/journal.pone.0217776)

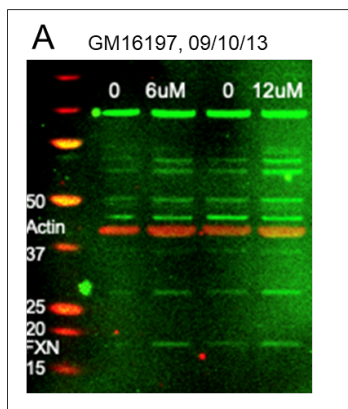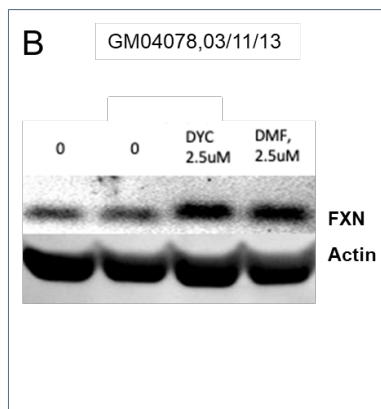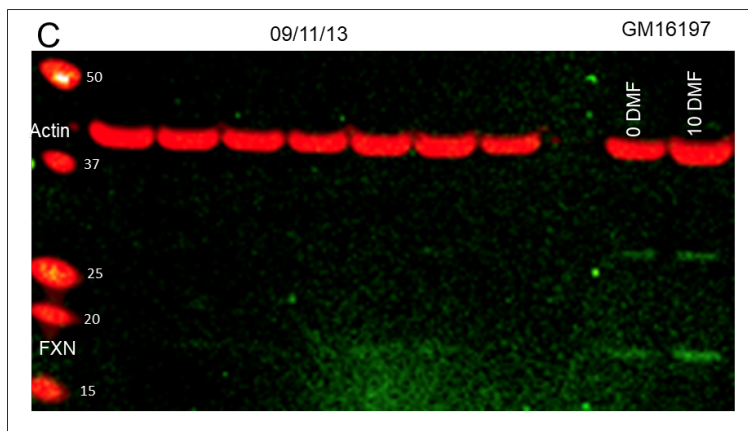

Supplement: S1 Fig — (PDF) [file pone.0217776.s003.pdf]

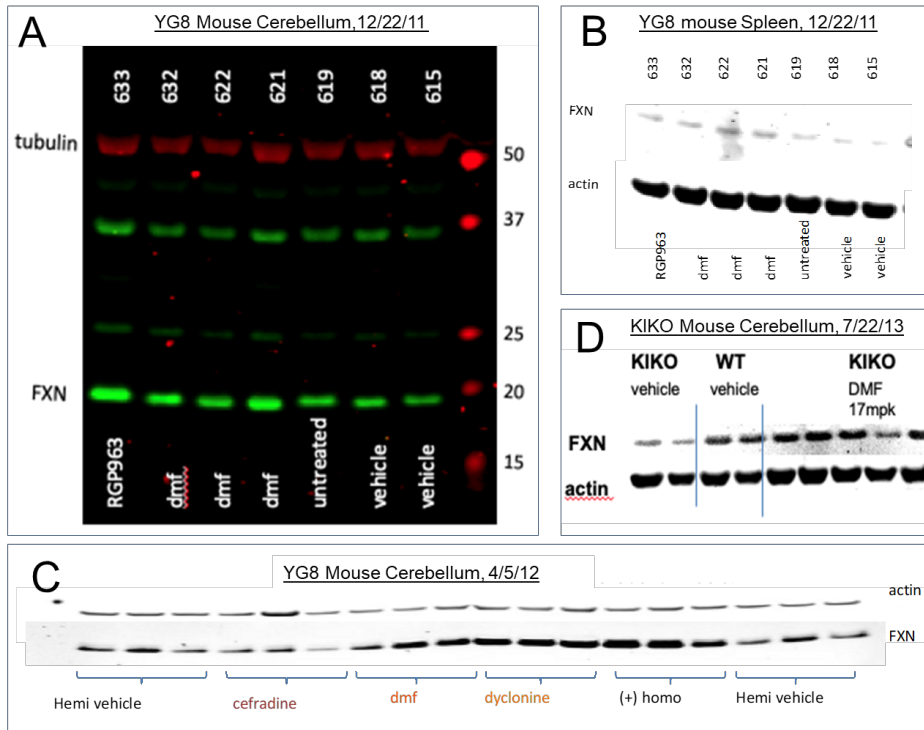

Supplement: S2 Fig — (PDF) [file pone.0217776.s004.pdf]

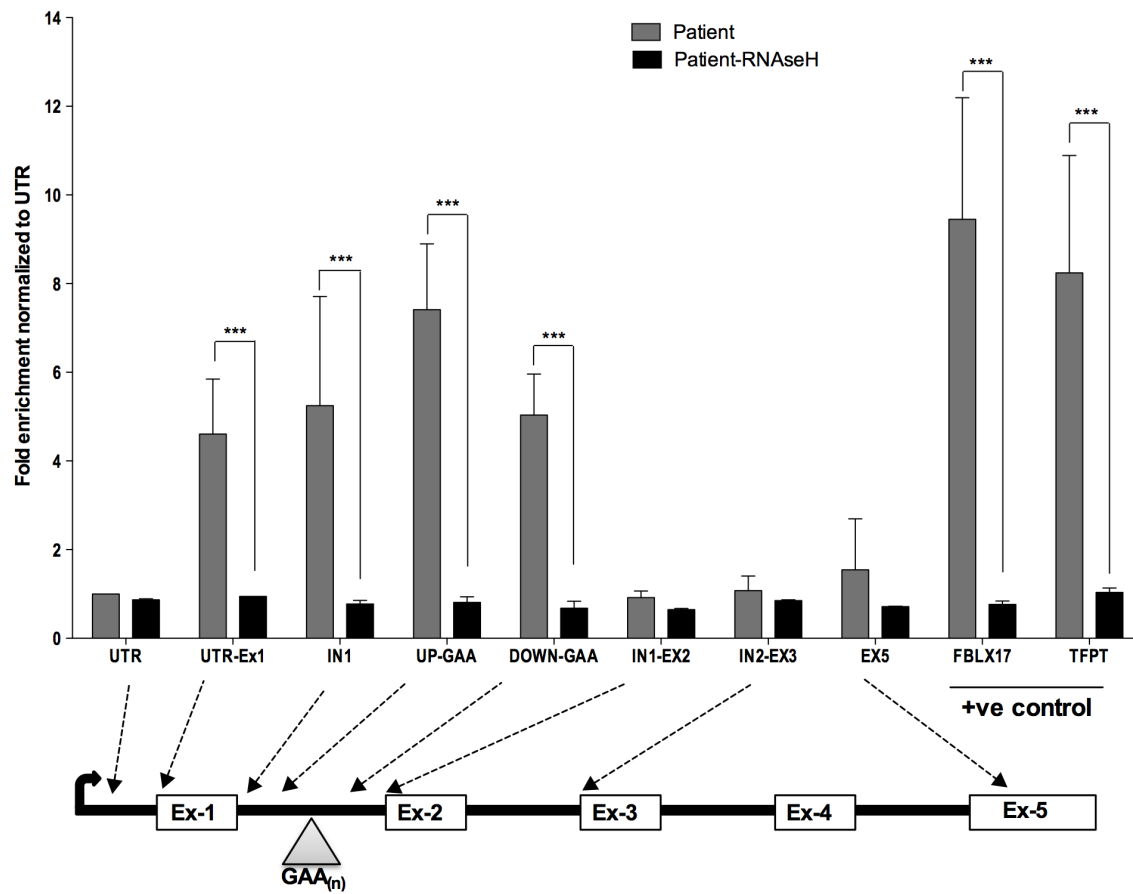

Supplement: S3 Fig — (PDF) [file pone.0217776.s005.pdf]
